# Genome-wide association studies of fertility and calving traits in Brown Swiss cattle using imputed whole-genome sequences

**DOI:** 10.1186/s12864-017-4308-z

**Published:** 2017-11-25

**Authors:** Mirjam Frischknecht, Beat Bapst, Franz R. Seefried, Heidi Signer-Hasler, Dorian Garrick, Christian Stricker, Intergenomics Consortium, Ruedi Fries, Ingolf Russ, Johann Sölkner, Anna Bieber, Maria G. Strillacci, Birgit Gredler-Grandl, Christine Flury

**Affiliations:** 1 Qualitas AG, Chamerstrasse 56a, 6300 Zug, Switzerland; 2 School of Agricultural, Forest and Food Sciences HAFL, Bern University of Applied Sciences, Länggasse 85, 3052 Zollikofen, Switzerland; 3 Institute of Veterinary, Animal & Biomedical Sciences, Massey University, Hamilton, New Zealand; 4 agn Genetics GmbH, 8b Börtjistrasse, 7260 Davos, Switzerland; 5 Interbull center, SLU - Box 7023, S-75007 Uppsala, Sweden; 6 Technische Universität München, Liesel-Beckmann-Straße 1, 85354 Freising-Weihenstephan, Germany; 7 Tierzuchtforschung e.V, Senator-Gerauer-Str. 23, 85586 Poing, Germany; 8 University of Natural Resources and Life Sciences, Gregor-Mendel-Str 33, 1180 Wien, Austria; 9 Research Institute of Organic Agriculture (FiBL), Ackerstrasse 113, 5070 Frick, Switzerland; 10 Department of Veterinary Medicine, University of Milan, Via Celoria 10, 20133 Milan, Italy

**Keywords:** Whole genome sequencing, Genome-wide association study, QTL discovery, Functional traits, Brown Swiss, Dairy cattle, Calving ease, Fertility

## Abstract

**Background:**

The detection of quantitative trait loci has accelerated with recent developments in genomics. The introduction of genomic selection in combination with sequencing efforts has made a large amount of genotypic data available. Functional traits such as fertility and calving traits have been included in routine genomic estimation of breeding values making large quantities of phenotypic data available for these traits. This data was used to investigate the genetics underlying fertility and calving traits and to identify potentially causative genomic regions and variants.

We performed genome-wide association studies for 13 functional traits related to female fertility as well as for direct and maternal calving ease based on imputed whole-genome sequences. Deregressed breeding values from ~1000–5000 bulls per trait were used to test for associations with approximately 10 million imputed sequence SNPs.

**Results:**

We identified a QTL on BTA17 associated with non-return rate at 56 days and with interval from first to last insemination. We found two significantly associated non-synonymous SNPs within this QTL region. Two more QTL for fertility traits were identified on BTA25 and 29. A single QTL was identified for maternal calving traits on BTA13 whereas three QTL on BTA19, 21 and 25 were identified for direct calving traits. The QTL on BTA19 co-localizes with the reported BH2 haplotype. The QTL on BTA25 is concordant for fertility and calving traits and co-localizes with a QTL previously reported to influence stature and related traits in Brown Swiss dairy cattle.

**Conclusion:**

The detection of QTL and their causative variants remains challenging. Combining comprehensive phenotypic data with imputed whole genome sequences seems promising. We present a QTL on BTA17 for female fertility in dairy cattle with two significantly associated non-synonymous SNPs, along with five additional QTL for fertility traits and calving traits. For all of these we fine mapped the regions and suggest candidate genes and candidate variants.

**Electronic supplementary material:**

The online version of this article (10.1186/s12864-017-4308-z) contains supplementary material, which is available to authorized users.

## Background

Inadequate fertility and problems associated with calving have high economic importance because they collectively cause 40% of the involuntary culling that occurs in the Brown Swiss population [1]. Calving ease is also an important because it influences animal welfare. Fertility and calving ease traits are included in routine national evaluations of several countries [2] including the Swiss dairy breeding programs [3, 4]. Genome-wide association studies (GWAS) for fertility and calving ease traits have been performed for several cattle breeds. For female fertility a number of quantitative trait loci (QTL) have been reported in different populations [5–7]. In Danish Jersey cattle the use of imputed whole-genome sequence data allowed the identification of various QTL influencing their national fertility index [8], which includes traits such as number of inseminations per conception, interval from calving to first insemination, 56-day non-return rate and days from first to last insemination. Those QTL were located on bovine chromosome (BTA) 7, 9, 20, 23, 25. Most variants found to have the highest significance of association with that fertility index were intergenic, except one missense variant associated with non-return rate on BTA23. In Italian Holstein, QTL have been identified using 50 K SNP chip data on BTA5, 7, 8, 13, 16, 18 and 27 for days to first service and on BTA2, 17 and 19 for their aggregate fertility index [9].

A locus on BTA18 has been associated with calving ease in Holstein [10]. Other QTL for calving ease have been identified in German Fleckvieh on BTA14 and 21 [5]. The QTL on BTA14 has been associated with stature in German Fleckvieh [11]. In Nordic Red cattle a QTL has been identified to be associated with a sire calving index that includes calving ease, stillbirth rate and a body conformation index including stature [12]. Further genomic associations with calving ease have been found on BTA2 in Limousin and Charolais beef breeds [6]. Overall these studies revealed breed specific loci, often located in regions that are also associated with stature.

Genomic information on thousands of progeny tested bulls is available today as a result of the introduction and now widespread use of genomic selection [13]. With the 1000 Bull Genomes Project [14, 15], a reference panel for imputation to sequence level has become available to project partners. Imputed whole-genome sequence data used in GWAS enhances the discovery of causative variants [16].

The objective of this study was to identify QTL affecting fertility and calving ease traits using imputed whole-genome sequence genotypes of Brown Swiss bulls. Furthermore we aimed to fine-map those QTL to identify potentially causative genes and variants.

## Methods

### Animals

A total of ~23,000 Brown Swiss animals genotyped at various densities were available from routine genomic prediction including those involving data sharing among the InterGenomics consortium [17] and the LowInputBreeds project (FP7-project no. KBBE 222623).

### Phenotypes

Estimated breeding values and corresponding reliabilities for five fertility traits were available: non-return rates for heifers (NRH) and cows (NRC) after 56 days; days to first service (DFS); interval between first and last insemination in heifers (IFLH) and in cows (IFLC) [4].

Deregressed breeding values for stillbirth (SB), calving ease (CE), gestation length (GL) and birth weight (BW) were available. For these four calving traits, a GWAS was carried out separately for maternal (m) and for direct (d) effects (Table 1). Additional analyses were undertaken for CEd (calving ease direct) with stature (s) as a covariate (CEd_sc) and for SBd and SBm with exclusion of some of the individuals (SBd corr; SBm corr). In total, breeding values for 13 traits were obtained from the Swiss Brown Swiss routine genomic evaluation [3, 4] and deregressed according to Garrick et al. [18]. We limited the analyses to breeding values of progeny tested bulls with reliabilities of estimated breeding values above 0.55 for fertility traits (except for DFS where the cutoff was 0.65) (Additional file 1: Figure S1) and above 0.20 for calving traits (Additional file 2: Figure S2). These thresholds were chosen to be the same as those used for choosing bulls to be included in the training set for routine genomic prediction. After these filters were applied, there were deregressed breeding values (deregBV) available for GWAS with 1136–4975 bulls depending upon trait (Table 1).

**Table 1:** Minimum, maximum and mean deregressed breeding value (deregBV) and genomic inflation factor lambda and number of individuals included in GWAS per trait

Trait	Min deregBV	Max deregBV	Mean (sd) deregBV	Genomic inflation factor (Lambda)	Number of individuals
NRH	−75.08	64.28	0.90 (15.68)	0.983	2506
NRC	−58.99	46.41	1.22 (14.67)	0.994	3615
IFLH	−62.74	50.40	−0.17 (15.34)	0.976	1484
IFLC	−52.94	46.50	−1.39 (13.75)	0.990	4122
DFS	−61.88	41.91	−2.69 (12.52)	0.990	3619
CEd	−210.01	121.45	−3.37 (17.93)	0.974	4975
CEd_sc	−210.01	121.45	−4.32 (17.36)	0.992	4159
SBd	−114.71	291.12	−13.00 (27.54)	1.010	1610
GLd	−121.55	156.02	0.39 (18.26)	0.935	2753
BWd	−110.60	209.64	−0.59 (19.69)	0.948	2561
CEm	−122.15	107.57	1.09 (25.09)	0.990	2862
SBm	−187.92	180.70	−8.84 (28.73)	1.005	1136
GLm	−113.34	132.43	−1.78 (23.51)	0.981	2756
BWm	−92.46	97.23	0.82 (18.92)	0.978	2683
SBd corr	−114.71	291.12	−14.28 (27.80)	1.014	1496
SBm corr	−187.92	180.70	−8.58 (29.66)	1.003	1051

Trait: *NRH* non-return rate in heifers, *NRC* non-return rate in cows, *IFLH* interval from first to last insemination in heifers, *IFLC* interval from first to last insemination in cows, *DFS* Days to first service, *CEd* calving ease direct, *CEd_sc* calving ease direct with stature deregressed breeding value (deregBV) as covariate, *SBd* stillbirth direct, *GLd* gestation length direct, *BWd* birth weight direct, *CEm* calving ease maternal, *SBm* stillbirth maternal, *GLd* gestation length maternal, *BWd* birth weight maternal, *SBd corr* Stillbirth direct, excluding the smaller cluster, *SBm corr*, Stillbirth maternal, excluding the smaller cluster

SNPs: number of SNPs considered for GWAS after filtering

Min deregBV: Minimum deregressed breeding value for the trait

Max deregBV: Maximum deregressed breeding value for the trait

Mean (sd) deregBV: Mean and standard deviation of the deregressed breeding value for the trait

### Imputation

We performed a two-step imputation as this has previously been shown to be more accurate than imputation directly to sequence density [19]. The first step included the imputation from 50 k single nucleotide polymorphism (SNP) chip data to high density (HD) SNP chip data using the software package FImpute with default parameter settings and pedigree information [20]. In the second step we imputed the HD SNPs to sequence density using Minimac with default settings [21] based on sequence variants from the 123 Brown Swiss (BSW) cattle that had been included in run 5 of the 1000 Bull Genomes Project [14, 15]. Only sequence SNPs and biallelic indels with a minor allele frequency (MAF) > 0.01 were imputed and only those with R-sq. > 0.1 in the .info file from Minimac were retained for GWAS (Table 1). R-sq. is an internally calculated value given by Minimac that reflects the imputation quality [21].

### Genome-wide association studies

Genome-wide association studies were conducted using the mixed model approach implemented in EMMAX [22]. We used the –Z option along with dosage data from imputation as genetic information. Individuals having a pedigree-based gene proportion of Original Braunvieh (OB) [23] > 0.3 were excluded from analysis as animals with high OB-gene proportion would create sub-structures in the population that otherwise mostly comprises individuals with little or no OB-gene proportion. The individual OB-gene proportion and reliability of deregBV were used as covariates in the model. The genomic relationship matrix was calculated for animals included in the analysis for each trait from HD SNP chip genotypes using GCTA [24]. The genomic relationship matrix was used in the mixed model fitted by EMMAX. We performed a principal component analysis (PCA) in R [25] using the princomp function and plotted the first and the second PC to visualize relatedness captured by the genomic relationship matrix. Using the R package wasim [26] we colored the individuals according to their deregBV. We filtered alleles with MAF < 0.05 and those deviating from Hardy-Weinberg equilibrium (HWE < 1 × 10^−20^). After filtering 9,748,130–9,999,287 variants were included in the GWAS. We used the Bonferroni corrected 0.05 significance threshold and for suggestive threshold we used *p* < 1 × 10^−6^.

Genomic inflation factor lambda was calculated in R applying the following formula:

$$ \mathrm{lambda}=\mathrm{round}\left(\mathrm{median}\left(\mathrm{qnorm}\left(\mathbf{p}/2\right)\hat{\mkern6mu} 2\right)/\mathrm{0.454,3}\right) $$

where **p** is the vector of *p*-values from the EMMAX GWAS.

### Variant annotation and description

All sequence variants were annotated using the Variant effect predictor (VEP) [27]. Frequency estimation of the sequence variants of interest were calculated within and across breeds using data from run 5 data of the 1000 Bull Genomes Project [14]. Linkage disequilibrium between variants was calculated using the --ld funcion in PLINK [28, 29]. We performed in silico prediction of the impact of missense variants using PolyPHEN2 [30]. Additionally the prediction of the impact of the variant from SIFT was available from VEP. Multiple sequence alignments were done using MAFFT (http://mafft.cbrc.jp/alignment/server/).

## Results and discussion

We performed GWAS for five fertility and eight calving traits using filtered imputed whole-genome sequence SNPs assuming that the causative variants were included in the data set. We investigated the QTL we identified for significantly associated variants, in terms of variants with direct impacts on proteins (e.g. missense or frame-shift mutations). Most traits we investigated have low heritability (exception GLd with 0.46) [3, 4]. We tried to keep our population relatively uniform by excluding animals with an OB-gene proportion > 0.3. The lambda values indicating genomic inflation (Table 1) reveal that for all traits except SBd and SBm, EMMAX tends to overcorrect for stratification (lambda < 1). In the PCA plot of the genomic relationship matrix for those individuals included for these two traits reveals two clusters (Additional file 3: Figure S3), while for the other traits the PCA plots show that the individuals are uniformly and continuously distributed with respect to the first two principal components (Additional file 3: Figure S3 and Additional file 4: Figure S4). Inspecting the relationships in the smaller cluster of the PCA plot from the individuals included in the GWAS for SB, we found one bull with about 90 male offspring as well as the sire of this bull. This heavily imbalanced relationship is likely to cause the substructure.

### Fertility traits

**Fig. 1:**
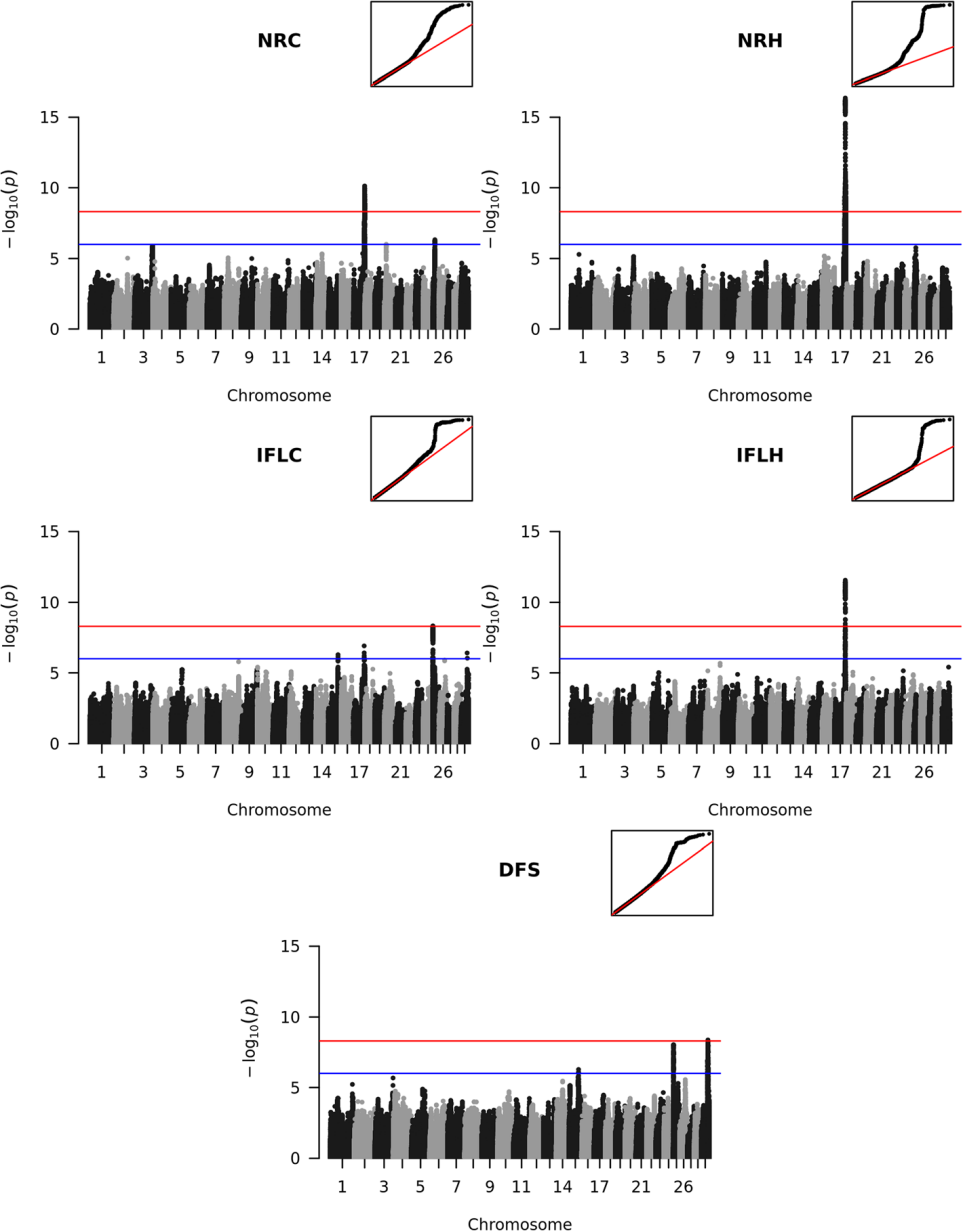
Manhattan plots for genome-wide association studies for fertility traits. The red line marks the Bonferroni corrected significance threshold. The blue line shows the threshold for suggestive variants. Small figures in the upper right corner show the qqplot of the *p*-values. NRC: Non-return rate in cows; NRH: Non-return rate in heifers; IFLC: Interval from first to last insemination in cows; IFLH: Interval from first to last insemination in heifers; DFS: Days to first service

We found significant associations for each of the five female fertility traits NRH, NRC, IFLH, IFLC and DFS (Fig. 1, Additional file 5: Table S1). The significant QTL were identified on BTA17 (NRH, IFLH and NRC), 25 (IFLC) and 29 (DFS). Beside the three significant associations of the QTL on BTA17, it was also suggestively associated with IFLC. Similarly, the QTL for IFLC on BTA25 was also suggestively associated with DFS. Additionally we found a suggestive QTL for DFS on BTA15.

**Fig. 2:**
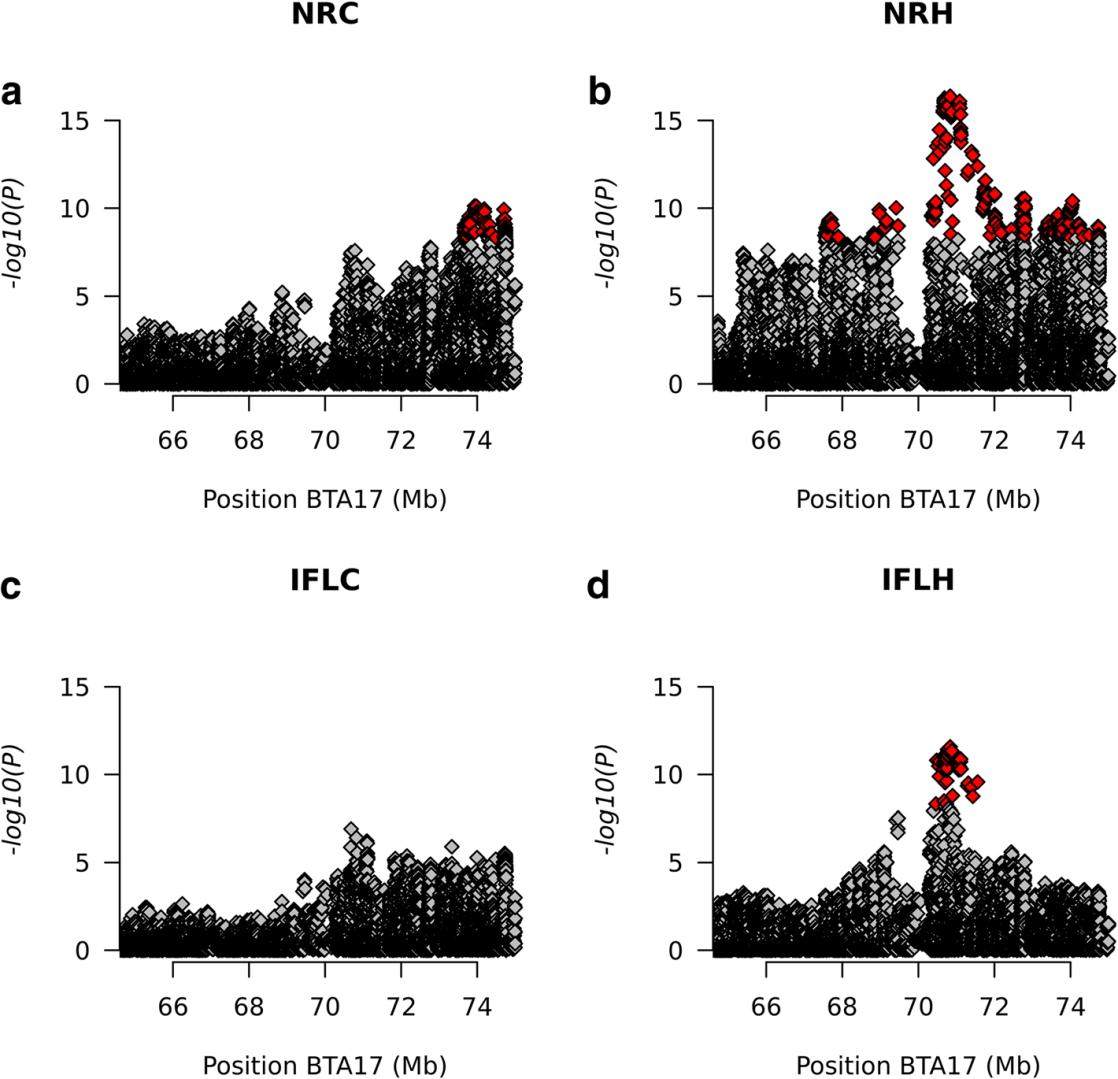
Association of variants with fertility traits on BTA17 from 66 to 75 Mb. The significantly associated variants are marked in red. **a** Non-return rate in cows (**b**) Non-return rate in heifers (**c**) Interval from first to last insemination in cows (**d**) interval from first to last insemination in heifers

The QTL on BTA17 is located at around 70–73 Mb. A zoom in this region reveals that the region with the most associated SNPs for IFLC, IFLH and NRH is not significantly associated with NRC (Fig. 2). For that trait, the peak is shifted about 3 Mb and is located around 73 Mb on the same autosome. We speculate that this is a second QTL, which is significant for NRC and NRH. For this QTL, we could identify two missense variants (Table 2). One of the missense variants is significantly associated with NRH and located in the *CABIN1* gene. That gene has been found to be significantly associated with fertility traits in Holstein [31] and also differentially expressed in cows from a high and a low fertility group, based on estimated breeding value for calving interval [32]. Even more interesting is the nonsense variant in *ENSBTAG00000048030*, also located in the 73 Mb QTL. Nonsense variants introduce a premature stop codon and are therefore unlikely to produce a functional protein. Using BLAST we found that the sequence of the corresponding protein has an identity of 84% with XP_015322696.1, which is encoded by the *IGLL1* gene on BTA17. That protein is implicated in immune-response and has been shown to be differentially expressed in the endometrium of cows that either showed or did not show signs of estrus around artificial insemination [33]. This gene may play a role in fertilization and therefore we propose the variant in the *IGLL1* gene as a candidate variant for fertility traits in dairy cattle. We think that the 73 Mb QTL is present in multiple breeds, such as Italian Holstein [9], while the peak around 71 Mb seems to be specific to the Brown Swiss breed in relation to fertility traits.

**Table 2:** Missense and nonsense variants that were significantly (*p* < 5*10^−9^) associated with fertility traits

Trait	BTA	Position	*p*-value	Gene	Variant	PolyPHEN2	SIFT
NRH	17	70,724,328	1.63 × 10^−16^	*GAS2L1*	p.P655L	unknown	deleterious (low confidence)
IFLH			2.20 × 10^−11^				
NRH	17	71,084,044	8.03 × 10^−17^	*ASCC2*	p.P42L	probably damaging	deleterious
IFLH			3.52 × 10^−11^				
NRH	17	72,747,746	8.78 × 10^−10^	*SLC5A4*	p.G608S^a^/p.G235S^b^	probably damaging	deleterious
NRH	17	72,815,579	1.61 × 10^−9^	*ENSBTAG00000048030*	p.Y108*	–	–
NRH	17	73,344,409	1.39 × 10^−9^	*CABIN1*	p.R651Q	benign	tolerated
NRH	17	73,393,194	1.09 × 10^−9^	*CABIN1*	p.A1721V	benign	tolerated (low confidence)
NRH	17	73,442,633	2.37 × 10^−9^	*ENSBTAG00000046900*	p.T234A	benign	tolerated
NRC	17	74,739,013	3.25 × 10^−9^	*CDC45L*	p.A263T	benign	tolerated
NRH			3.49 × 10^−9^				

Trait: *NRC* non-return rate in heifers, *NRH* non-return rate in heifers, *IFLH* interval from first to last insemination in heifers

BTA: Chromosome

*P*-value: From GWAS with EMMAX

Variant: Amino acid change caused by the variant

PolyPhen2: Predicted effect of the variant on the protein function from PolyPhen2

SIFT: Predicted effect of the variant on the protein function from SIFT obtained via Variant effect predictor (VEP)

^a/b^The transcripts ENSBTAT00000010678^a^ and ENSBTAT00000052915^b^ for *SLC5A4*

There is additional evidence that the genomic regions of these QTL might affect quantitative traits in the Brown Swiss population because two complex copy number variant (CNV) regions have been found to be located at 72–73 Mb and 73–75 Mb [34] using the consensus map of CNV detected from PennCNV and SVS algorithms. A similar region for the Mexican Holstein breed was reported by Duran Aguilar et al. [35] comprising three CNV regions from 70.7 Mb to 70.9 Mb mapped using PennCNV. This latter study also reports a significant association with SCS, a functional trait under selection in most of the dairy cattle populations.

The peak region for IFLH is located between 70,462,351 bp and 71,559,004 bp on BTA17, which is the center of the 71 Mb QTL. The 70 most associated SNPs of NRH are within this same interval. In this interval two missense variants can be identified to be significantly associated in the GWAS and in the sequenced Brown Swiss animals they are in perfect LD with the top associated variant from the GWAS. The two variants are located in the *GAS2L1* gene (g.70,724,328C > T) and in the *ASCC2* gene (g.71,084,044G > A).

**Fig. 3:**
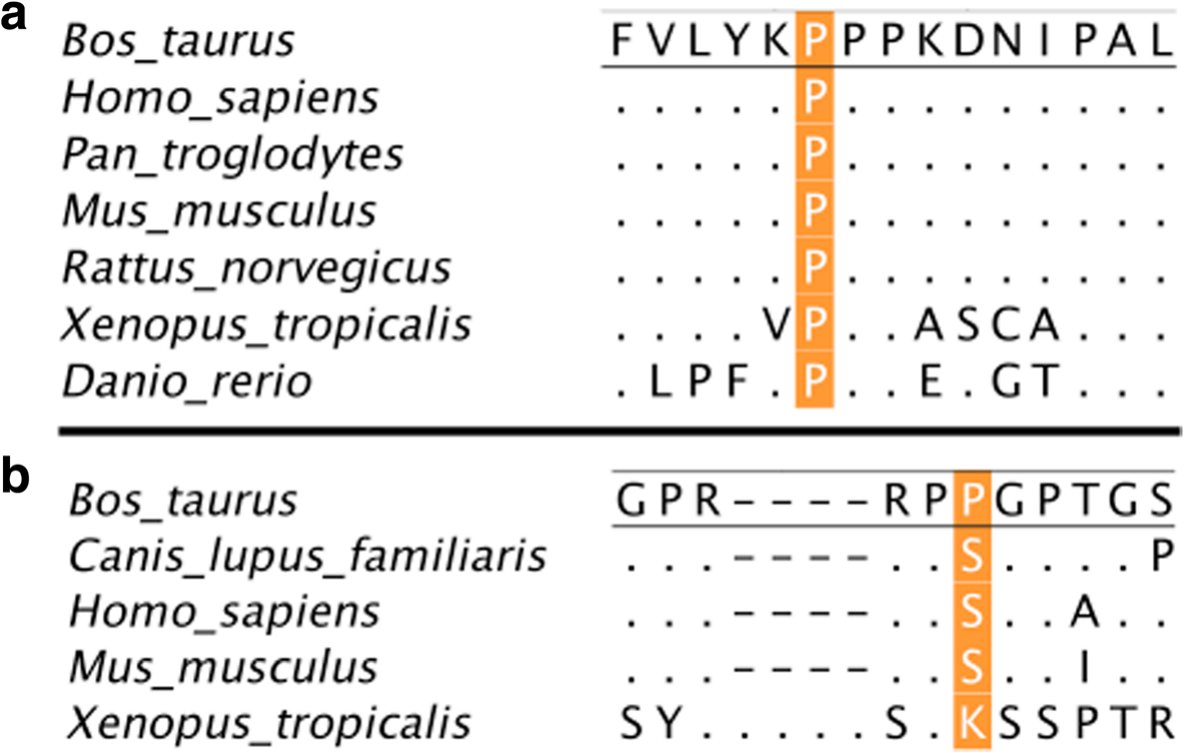
Multiple sequence alignment of (**a**) ASCC2 (amino acids 37–51 (*Bos taurus*)) the orange shading marks the p.42 position. The sequences for ASCC2 were derived from the following accession numbers: *Bos taurus*, NP001015524.1; *Homo sapiens*, NP_115580.2; *Pan troglotydes*, XP_515064.3; *Mus musculus*, NP_083567.1; *Rattus norvegicus*, NP_001102561.1; *Xenopus tropicalis*, NP_001016871.1; *Danio rerio*, NP_956736.1 and (**b**) GAS2L1 (amino acids 650- the orange shading marks the p.655 position. The sequences for GAS2L1 were derived from the following accession numbers: *Bos taurus*, NP_001077167.1; *Canis lupus familiaris*, XP_543468.2; *Homo sapiens*, NP *Mus musculus*, NP_653146.1; Xenopus tropicalis, XP_002934334.1

For both variants the alternative allele has a negative effect on the deregBV. Using in silico effect prediction on the protein, SIFT (provided by VEP) and PolyPHEN2 revealed that the variant in *ASCC2* (p.P42L) is likely to have deleterious (0.02 - SIFT) or probably damaging (0.992 - PolyPHEN2) impact. The effect of the variant in GAS2L1 (p.P655L) could not reliably be predicted (deleterious low confidence (0.02); unknown). The variant in *ASCC2* is specific to Brown Swiss cattle. In the run 5 of the 1000 Bull Genomes Project, 22 Brown Swiss were found to be heterozygous for this variant whereas 1 animal was homozygous (Additional file 6: Table S2). Additionally we found the variant in two Danish Red cattle, however according to their pedigree, both are sired by animals with breed code BSW. The MAF of this variant is close to 10% in BSW, while below 1% across all the sequenced individuals of different breeds. The *GAS2L1* p.P655L variant was not only found in BSW but also in Simmental, Angus, Jersey and Hereford. Due to the high LD between these two variants we speculate that the *GAS2L1* variant is older and the *ASCC2* variant occurred in Brown Swiss on the haplotype carrying the *GAS2L1* variant. Since we found homozygous individuals in the data set of sequenced animals for both variants, neither of the variants can be homozygous lethal. *ASCC2* is involved in gene activation and repression as part of the ASC1 complex. In order to have support of the hypothesis that the p.P42L variant in ASCC2 is more likely the causative mutation rather than *GAS2L1* variant. We performed multiple sequence alignments for both p.P42L in *ASSC2* and p.P655L in *GAS2L1* (Fig. 3). In ASCC2 the amino acid at this position is conserved among all mammals in our comparison and in other species down to the zebra fish. The proline at position 655 in *GAS2L1* on the other hand is not conserved at all.

The locus on BTA25 is associated with IFLC. This region on BTA25 has been associated with stature in Brown Swiss cattle [36] and we found it to be associated with calving ease (see below). In Brahman cows it has been shown that larger cattle had lower pregnancy rates [37]. That author suggests the lower pregnancy rate is mainly due to negative energy balance in lactating cows, which cannot be compensated by increased feed intake [37]. This might explain why we do not find this locus to be associated with IFLH, the same fertility trait in heifers, which are not lactating when they become pregnant. For DFS, which is also a trait only measurable in lactating cows, we found a suggestive association for this locus. In a previous study in Brown Swiss this locus has been shown to be associated with a fertility trait (cows ability to recycle after calving) and other traits [36].

The second QTL we identified for a single trait was on BTA29 at around 44 Mb and was associated with DFS. The only significantly associated variant is located in an intron of *PYGM*. *PYGM* encodes myophosphorylase, which is a glycogen phosphorylase expressed in muscle. Non-synonymous variants in this gene have been shown to cause protein alterations involved in glycogen storage disease type V [38]. It remains unclear how this gene could be related to fertility. In the QTL region on BTA29 a second plausible candidate gene is located, which is *PLCB3*. That gene has been found to be differentially expressed in low and high fertility Holstein cows [32]. Among the significantly associated variants, none were located in this gene. However there is a suggestively associated synonymous variant in *PLCB3*. Synonymous variants can influence translation efficiency if a rare codon is used [39]**,** which would subsequently influence the level of gene expression and amount of protein available. Since there is differential expression in low and high fertility Holstein cows a lower translation rate and a subsequent lower amount of protein for PLCB3 could potentially impact fertility.

### Calving traits

**Fig. 4:**
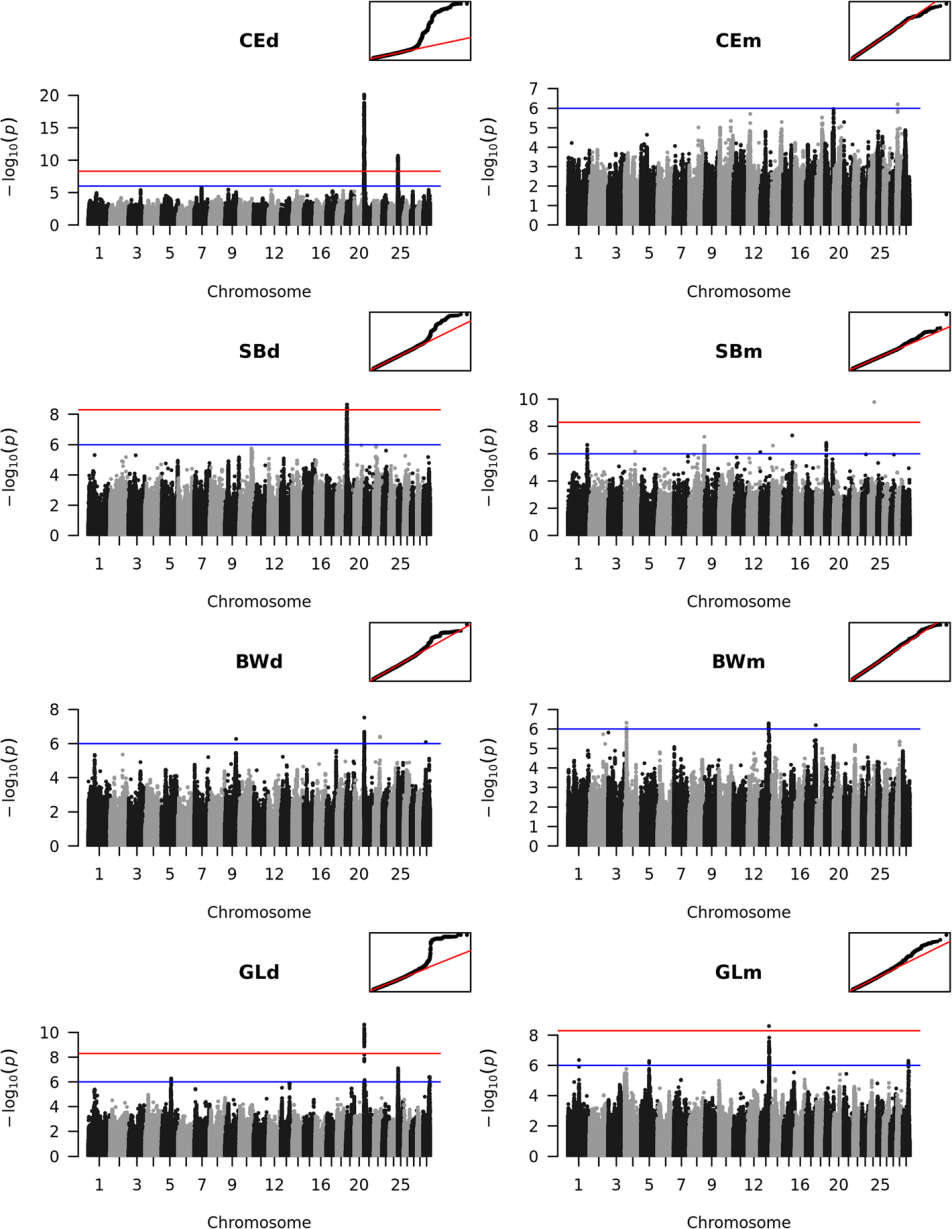
Manhattan plots for the genome-wide association studies for calving traits. The red line marks the Bonferroni corrected significance threshold. The blue line shows the threshold for suggestive variants. Small figures in the upper right corner show the corresponding qqplot. CEd: Calving ease direct; CEm: Calving ease maternal; SBd: Stillbirth direct; SBm: Stillbirth maternal; BWd: Birth weight direct BWm: Birth weight maternal; GLd: Gestation length direct; GLm: Gestation length maternal

For the direct calving traits we could identify significantly associated regions for the three traits CEd, SBd and GLd (Fig. 4, left side, Additional file 5: Table S1). Only for BWd no locus was found to be significantly associated with the trait. Unlike for the fertility traits, we found different QTL for each calving trait. Those QTL with significant association were located on BTA19, 21 and 25. Additionally we identified suggestive QTL on BTA5, 22 and 29.

#### Direct calving ease and gestation length

For direct calving ease (CEd) two QTL were identified, on the proximal ends of BTA21 and BTA25. The locus on BTA25 has been associated with stature in Brown Swiss cattle [36]. Intuitively, it seems logical that large calves would determine more birth difficulties than small calves when born to cows of the same stature. However genetic correlations between stature and calving ease are non-significant in UK Holstein-Friesian cattle [40]. We also found only a low correlation between those two traits of 0.1 for the phenotypic correlation of the deregressed breeding values of the two traits. This might be explained by the fact that not all loci influencing adult size have an impact on the size of the calf. However, we additionally performed GWAS for calving ease using the deregressed breeding value for stature as a covariate. With stature as a covariate the signal on BTA25 disappears. (Additional file 7: Figure S5). We interpret, that the locus on BTA25 is either pleiotropic or that the phenotypes observed are indeed due to differences in the size of the calves, with small calves being delivered more easily than tall calves. Even though this QTL has been described previously, no possible causative variant has been identified. For CEd, we found three missense variants among the significantly associated variants on BTA25, located in *CRAMP1L*, *PTX4* and *TELO2* genes (Table 3).

**Table 3:** Missense and nonsense variants that were significantly (*p* < 5*10^−9^) associated with calving traits

Trait	BTA	Position	*p*-value	Gene	Variant	PolyPHEN2	SIFT
CEd	25	1,277,577	5.59 × 10^−10^	*CRAMP1L*	p.R885Q	probably damaging	deleterious
CEd	25	1,177,995	1.93 × 10^−9^	*TELO2*	p.R536W	probably damaging	deleterious
CEd	25	1,161,904	4.05 × 10^−9^	*PTX4*	p.P207L	probably damaging	tolerated

Trait: *CEd* calving ease direct

BTA: Chromosome

*P*-value: From GWAS with EMMAX

Variant: Amino acid change caused by the variant

PolyPhen2: predicted effect of the variant on the protein function from PolyPhen2

SIFT: predicted effect of the variant on the protein function from SIFT obtained via Variant effect predictor (VEP)

* : Translation stop codon

All three variants were significantly associated with stature in BSW as well as CEd. The direction of the SNP effects was however opposite. According to PolyPHEN2 all three variants are probably damaging and therefore potentially causal. As for regions on BTA17, the CNVR_510 reported by Prinsen et al. [34] on BTA25 in BSW spans these significantly associated missense variants. The genomic variation CNVR_510 is detected in 86 individuals indicating that the CNV in this location may represent an important source of variation that may affect quantitative traits.

The locus on BTA21 remained significantly associated with calving ease when including stature as a covariate (Additional file 7: Figure S5). Therefore this locus is likely not involved in normal variation in adult stature. Interestingly the same locus had been identified earlier in German Fleckvieh cattle to be associated with the complex of calving traits [5]. However in that study an association of SB with the same QTL was reported, whereas we could not detect any association with SB in Brown Swiss. Moreover, we found this region to be associated with gestation length. Generally a longer gestation length is associated with higher birth weight and a subsequent increase in calving difficulties [3].

The direction of the SNP effects within the QTL on BTA 21 highlight this relationship between gestation length and calving ease. The mechanism by which this region could impact calving ease is unknown. It is known that the shared syntenic region in human is imprinted and associated with Prader-Willi syndrome for defects in the paternal allele and with Angelman syndrome for the maternal allele [41]. Human babies with Prader-Willi syndrome have an increased risk of being born by cesarean section [42], which would be in agreement with a decreased breeding value for calving ease. However on the other hand a further feature of Prader-Willi syndrome is that offspring are at increased risk of preterm birth, which does not match the expectation and known relationship between GL and CE.

We found 7 synonymous variants significantly associated with *ATP10A*. The *ATP10A* gene is maternally imprinted in human and associated with Angelman syndrome [43]. However, there is some evidence that this gene is not imprinted in mice [44]. We found suggestively associated non-synonymous variants in *ATP10A* and *MAGEL2.* The one located in the *ATP10A* gene, causes p.M655 L, predicted by SIFT to be tolerated and by PolyPHEN2 to be benign. The other variant causes an amino acid exchange from Glutamic acid to Glycine at the 851 amino acid position. For this variant no prediction about its effect size could be done. *MAGEL2* is implicated in Prader-Willi syndrome [45], which phenotypically seems to have a larger influence on birth traits than Angelman syndrome. For *MAGEL2* in bovine parthenogenic and normal embryonic cells no difference in the expression pattern could be detected suggesting this gene is not imprinted in cattle [46]. For mice that are heterozygous for *MAGEL2* deficiency, an increased perinatal lethality has been described [47].

For GLd, the top associated variants are about 800 kb downstream compared to those for CEd. The SNPs in this region are also significantly associated with CEd. When analysing the SNP density in this region we found that there is a decreased SNP density at the beginning of BTA21 (less than 1000 SNPs per Mb) relative to other locations. This observation is displayed in the zoomed plot (Additional file 8: Figure S6). The SNP density is similar for both traits in this region. We speculate that the causative variant is indeed the same for both traits.

#### Stillbirth

**Fig. 5:**
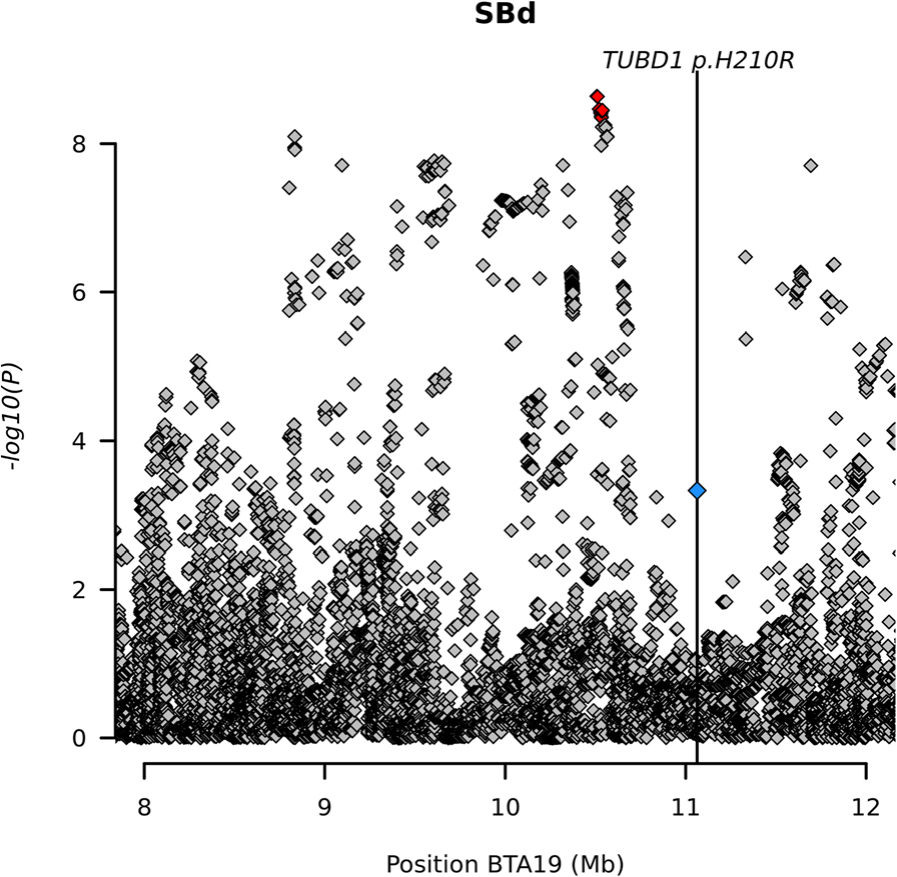
Association of stillbirth (direct) on BTA19 and location of *TUBD1* variant causing the BH2 phenotype. The significantly associated variants are marked in red

We identified several SNPs on BTA19 with significant associations with SB at around 10 Mb. That locus co-localizes with the BH2 haplotype for which a causative mutation has been identified in the *TUBD1* gene [48]. Calves homozygous for the *TUBD1* variant show high mortality rates during or shortly after birth. In our data set the MAF of this variant was lower than 0.05 and therefore it was not included in the GWAS, and in any event the imputation R-sq. was rather low (0.12) for this variant. However, because this variant is an obvious candidate causative variant for this gene we ran an additional single SNP regression for this variant generating a non-significant *p*-value of 4.68 × 10^−4^. Interestingly having a closer look at the *p*-values in the BH2 haplotype region, there were reduced –log_10_(p-values) observed compared to slightly upstream and downstream from the haplotype (Fig. 5). We have to remember that the model assumption for which association testing is performed is an additive mode of inheritance, but BH2 is never present in homozygous form. It is possible that another QTL is responsible for the signal. In order to verify that the association is truly caused by BH2, we removed BH2 carriers in an additional GWAS and in a second step we used BH2 status as a covariate. In both cases no significant SNP remained on BTA19. Therefore we conclude that the signal is indeed caused by BH2 (Additional file 9: Figure S7).

#### Maternal traits

We also performed GWAS for the maternal calving traits CEm, SBm, BWm and GLm (Fig. 4, right side, Additional file 5: Table S1). We found one SNP significantly associated with SBm. That SNP has an imputation R-sq. of 0.17 and MAF of 0.051. These values are only slightly above the quality control thresholds and therefore we believe that the significant effect of this SNP may represent a false positive. This is corroborated by the fact that no other SNP in that region shows either significant or suggestive association. There is a substructure in the individuals used for SBm and we believe this false-positive association may be due to that (Additional file 3: Figure S3 C and D). To support this hypothesis, we reran the GWAS excluding all individuals in the smaller cluster of the PCA plot (the sire with 90 offspring, its father and its siblings). The Manhattan plot clearly demonstrates that there is no longer any significantly associated SNP (Additional file 10: Figure S8). Because a reduction in the number of individuals also leads to a reduction of power to detect true associations, we removed the same individuals for SBd, and there the peak on BTA19 remained significant. These results show how important a good correction and an eventual exclusion of outlier individuals is in order to avoid false-positive findings.

**Fig. 6:**
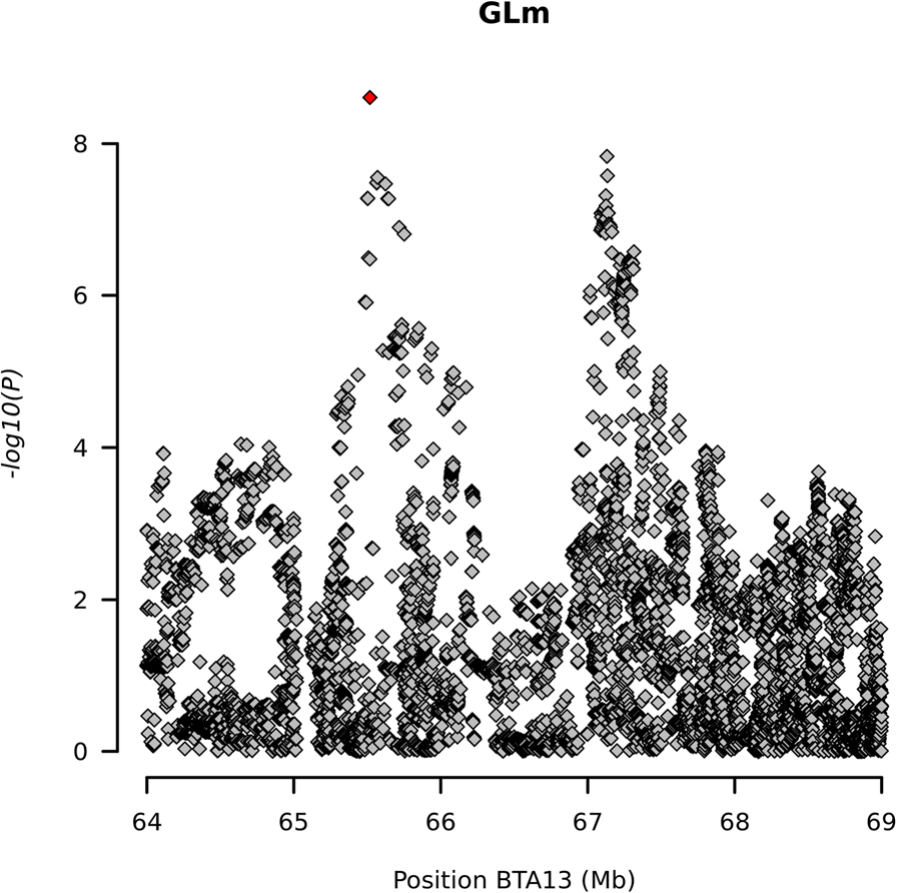
Association of maternal gestation length on BTA13. The significantly associated SNP is marked in red

The other maternal trait for which we found a significant association was GL, the association for GLm is only due to a single significant SNP but a number of nearby SNPs are suggestively associated, just below the significance threshold. Additionally this SNP has a higher imputation R-sq. (0.781) and MAF (0.383). The QTL for GLm was located on BTA13. Zooming on this region reveals that there could actually be 2 QTL: at 65.5 Mb with the significant SNP and a suggestive QTL at 67.1 Mb (Fig. 6). The two loci are not in strong LD (r^2^ = 0.35), supporting the hypothesis of two QTL in this region. The significantly associated variant at around 65.5 Mb, is located in an intron of *CPNE1*. *CPNE1* encodes a calcium-dependent phospholipid binding protein but little is known about the exact role of the protein. Recently it has been found that this gene is expressed in human placenta [49] but not found to be expressed above 50 fragments per kilobase million (FPKM) in the cattle transcriptome [49]. However the placentas used in that study came from term births and therefore differential expression over gestation cannot be ruled out. Totally 142 suggestively associated variants are distributed on the two QTL. Of these 15 are associated with the first QTL and include a missense variant in the *SPAG4* gene. The variant is p.Y289F and is predicted to be tolerated by SIFT, but PolyPHEN2 predicts that the variant is probably damaging. *SPAG4* encodes the sperm-associated antigen protein 4. As the name indicates, disruption of this gene leads to abnormal sperm development and decreased male fertility [50]. Additionally this gene has also been found to be differentially expressed in pre-eclamptic placenta in humans [51]. This gene is an interesting candidate gene for GLm.

At the second locus 127 variants are suggestively associated. Among these variants we find a single synonymous variant in the *CTNNBL1* gene. This gene has been found to be expressed in the placenta of *Ateles fusciceps* but not with FPKM > 50 in cattle. Deficiency of *CTNNBL1* in mice leads to embryonic lethality [52]. More interestingly for GLm are the genes *NNAT* and *BLCAP.* The peak of this second QTL is located between these two genes. Both genes are imprinted and expressed in the placenta [53]. Therefore these two genes provide good candidate genes for a maternal trait. We cannot suggest a possible causative variant. Variants with influence on gene expression but with no alteration of the protein are hard to detect. It is likely that the causative variant is located outside the coding region.

## Conclusion

In this study we used readily available phenotypes that are collected for routine genomic prediction to identify QTL for traits related to fertility and calving ease. We detected a novel QTL for fertility in Brown Swiss on BTA17 and suggest a candidate that may represent the causal variant. We further identified regions associated with other birth and fertility traits. We detected a region with a known deleterious variant to be significantly associated with stillbirth and a region associated with maternal gestation length including genes with placental expression in that associated region. This study gives insight into the genetic architecture of the functional traits that characterize fertility and calving ease.

## Additional files

Additional file 1: Figure S1. Distribution of reliabilities of estimated breeding values for fertility traits. NRC: Non-return rate in cows; NRH: Non-return rate in heifers; IFLC: Interval from first to last insemination in cows; IFLH: Interval from first to last insemination in heifers; DFS: Days to first service. (PNG 51 kb)

Additional file 2: Figure S2. Distribution of reliabilities of estimated breeding values for calving traits. CEd: Calving ease direct; CEm: Calving ease maternal; SBd: Stillbirth direct; SBm: Stillbirth maternal; BWd: Birth weight direct BWm: Birth weight maternal; GLd: Gestation length direct; GLm: Gestation length maternal. (PNG 57 kb)

Additional file 3: Figure S3. The first two principal coponents for the genomic relationship matrix comprising individuals used for genome-wide association of calving traits (A) Calving ease direct (B) Calving ease maternal (C) Stillbirth direct (D) Stillbirth maternal (E) Birth weight direct (F) Birth weight maternal (G) Gestation length direct (H) Gestation length maternal. (PNG 816 kb)

Additional file 4: Figure S4. The first two principal coponents for the genomic relationship matrix comprising individuals used for genome-wide association of fertility traits (A) Non-return rate in cows (B) Non-return rate in heifers (C) Interval from first to last insemination in cows (D) Interval from first to last insemination in heifers (E) Days to first service. (PNG 677 kb)

Additional file 5: Table S1. GWAS results per trait. SNPs with *p*-values < 10^−6^ for each trait. (XLSX 415 kb)

Additional file 6: Table S2. Minor allele frequency per breed among sequenced individuals for *ASCC2* and *GAS2L1*. (XLSX 44 kb)

Additional file 7: Figure S5. GWAS for calving ease using stature as a covariate. The red line marks the Bonferroni corrected significance threshold. The blue line shows the threshold for suggestive variants. The figure in the upper right corner shows the corresponding qqplot. (PNG 74 kb)

Additional file 8: Figure S6. Association for calving ease direct (CEd) and gestation length direct (GLd) on BTA21 from 1 to 4 Mb. The red line indicates the Bonferroni corrected significance threshold. (PNG 582 kb)

Additional file 9: Figure S7. Association on BTA19 for stillbirth using only individuals not carrying BH2 (top) or using BH2 carrier status as a covariate (bottom). The red line indicates the Bonferroni corrected significance threshold. (PNG 563 kb)

Additional file 10: Figure S8. Manhattan plots for the GWAS for stillbirth direct and maternal excluding individuals from the smaller cluster in the PCA plot. The red lines mark the Bonferroni corrected significance threshold. The blue lines show the threshold for suggestive variants. Small figures in the upper right corner shows the corresponding qqplot. (PNG 506 kb)

## Abbreviations

BSW: Brown Swiss Cattle
BTA: Bovine chromosome
BWd: Birth weight direct
BWm: Birth weight maternal
CEd: Calving ease direct
CEm: Calving ease maternal
CNV: Copy number variant
deregBV: Deregressed breeding value
DFS: Days to first service
FPKM: Fragments per kilobase million
GLd: Gestation length direct
GLm: Gestation length maternal
GWAS: Genome-wide association study
HD: High density
IFLC: Interval from first to last insemination in cows
IFLH: Interval from first to last insemination in heifer
MAF: Minor allele frequency
NRC: Non-return rate in cows
NRH: Non-return rate in heifers
PCA: Principal component analysis
QTL: Quantitative trait locus/loci
SBd: Stillbirth direct
SBm: Stillbirth maternal
SNP: Single nucleotide polymorphism
VEP: Variant effect predictor
