# Supplementary figures and images for: Genome-wide association studies of fertility and calving traits in Brown Swiss cattle using imputed whole-genome sequences
Source: BMC Genomics. 2017 Nov 25;18:910. doi: 10.1186/s12864-017-4308-z (PMC5702100; doi:10.1186/s12864-017-4308-z)

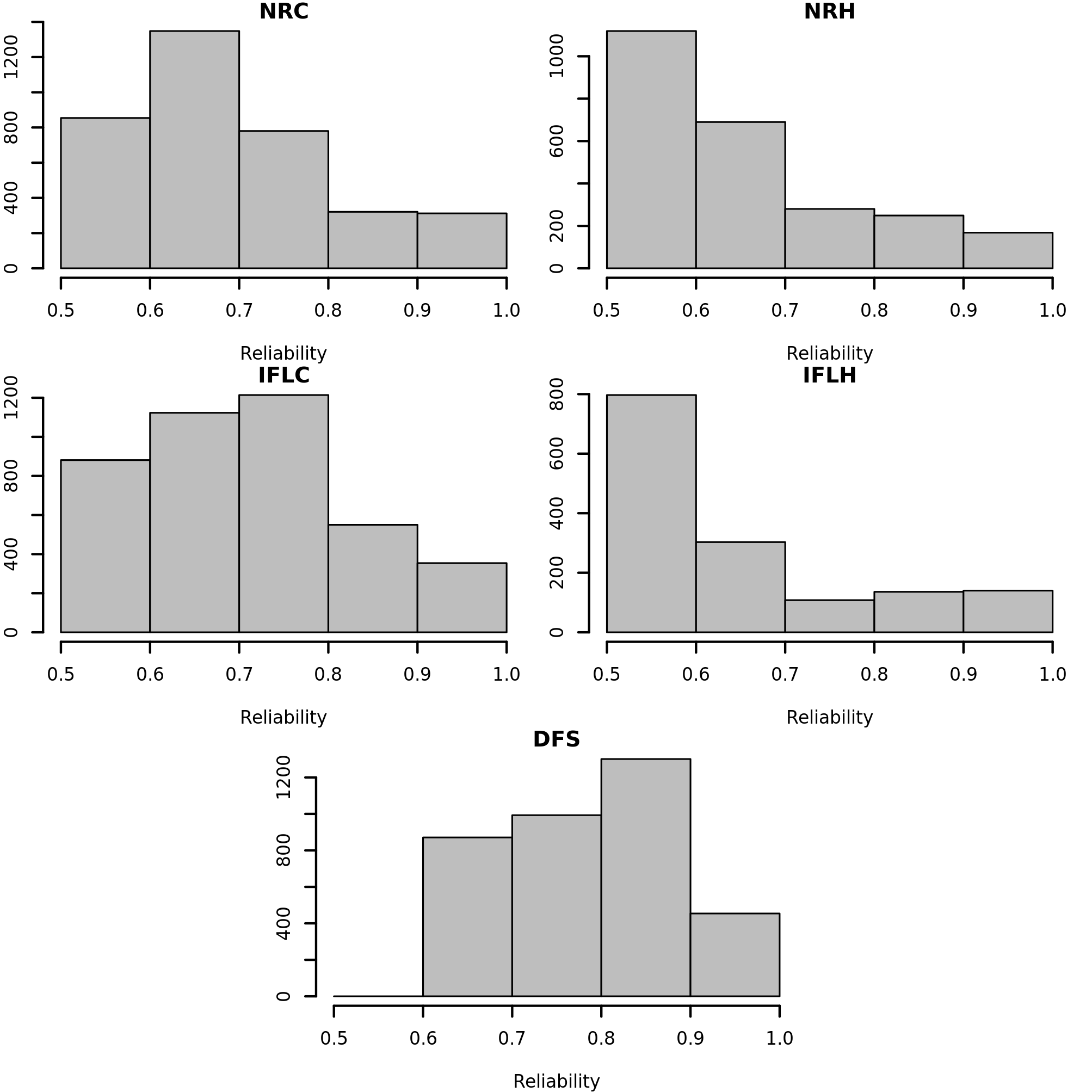

Supplement: Supplementary file 1 — Distribution of reliabilities of estimated breeding values for fertility traits. NRC: Non-return rate in cows; NRH: Non-return rate in heifers; IFLC: Interval from first to last insemination in cows; IFLH: Interval from first to last insemination in heifers; DFS: Days to first service. (PNG 51 kb) [file 12864_2017_4308_MOESM1_ESM.png]

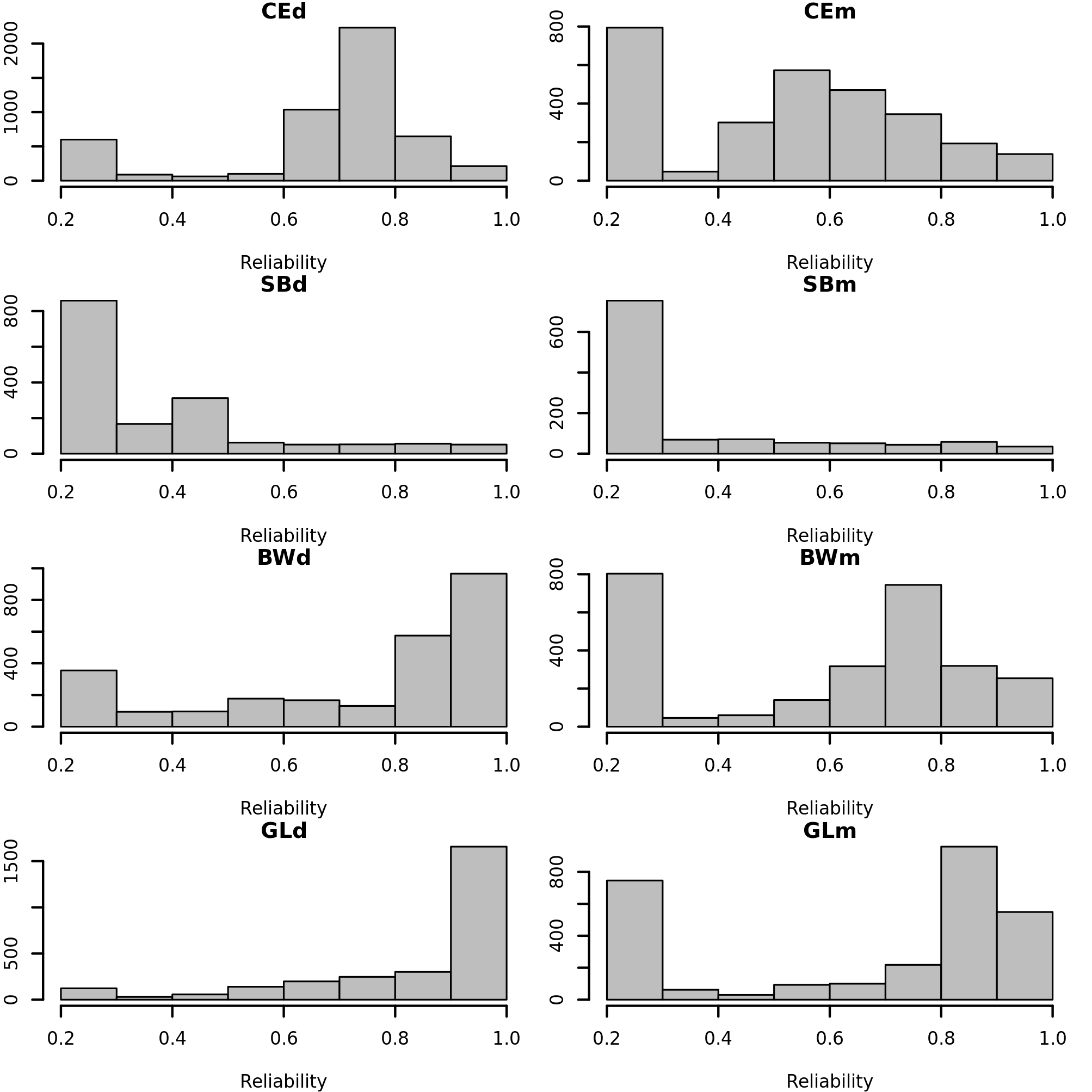

Supplement: Supplementary file 2 — Distribution of reliabilities of estimated breeding values for calving traits. CEd: Calving ease direct; CEm: Calving ease maternal; SBd: Stillbirth direct; SBm: Stillbirth maternal; BWd: Birth weight direct BWm: Birth weight maternal; GLd: Gestation length direct; GLm: Gestation length maternal. (PNG 57 kb) [file 12864_2017_4308_MOESM2_ESM.png]

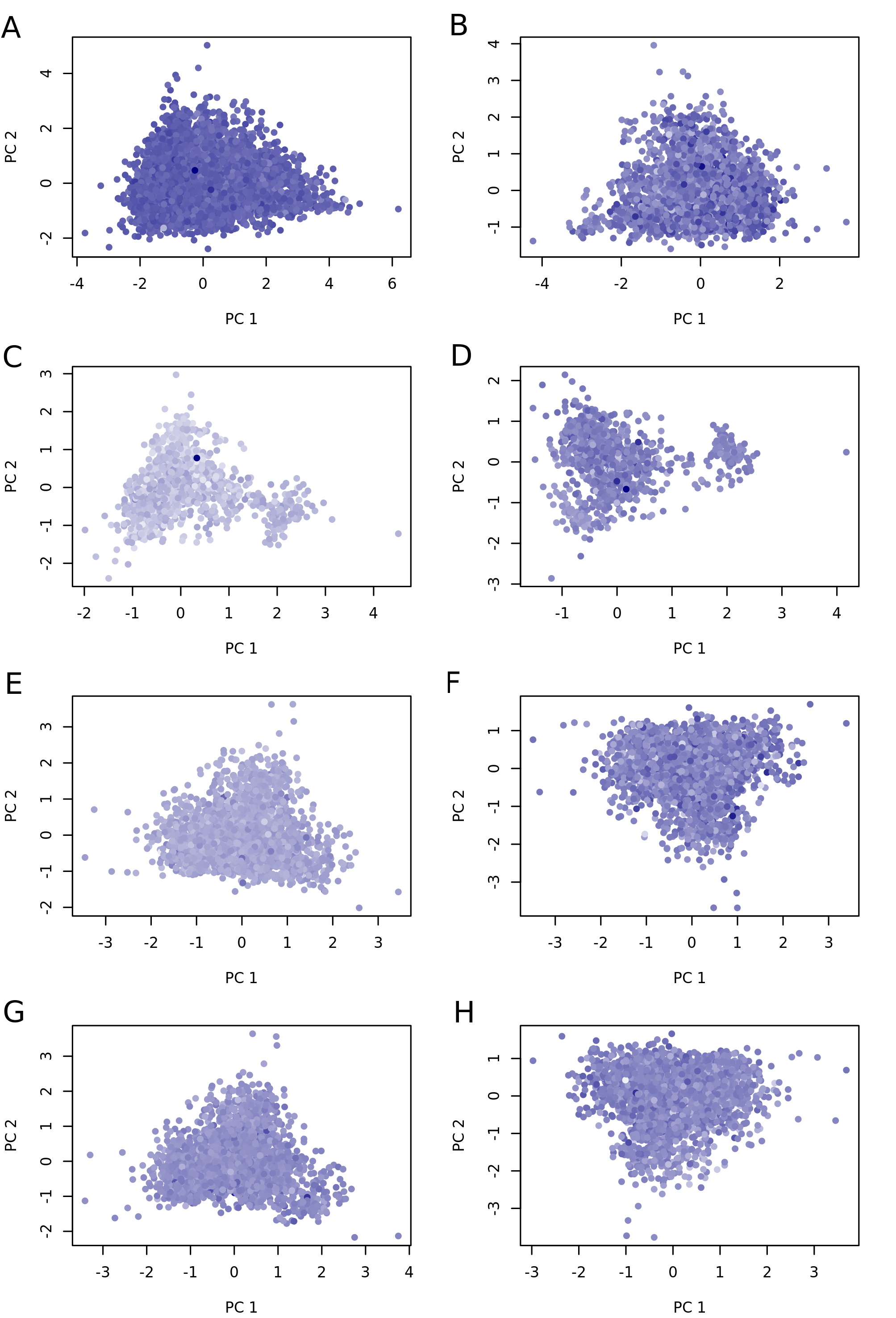

Supplement: Supplementary file 3 — The first two principal coponents for the genomic relationship matrix comprising individuals used for genome-wide association of calving traits (A) Calving ease direct (B) Calving ease maternal (C) Stillbirth direct (D) Stillbirth maternal (E) Birth weight direct (F) Birth weight maternal (G) Gestation length direct (H) Gestation length maternal. (PNG 816 kb) [file 12864_2017_4308_MOESM3_ESM.png]

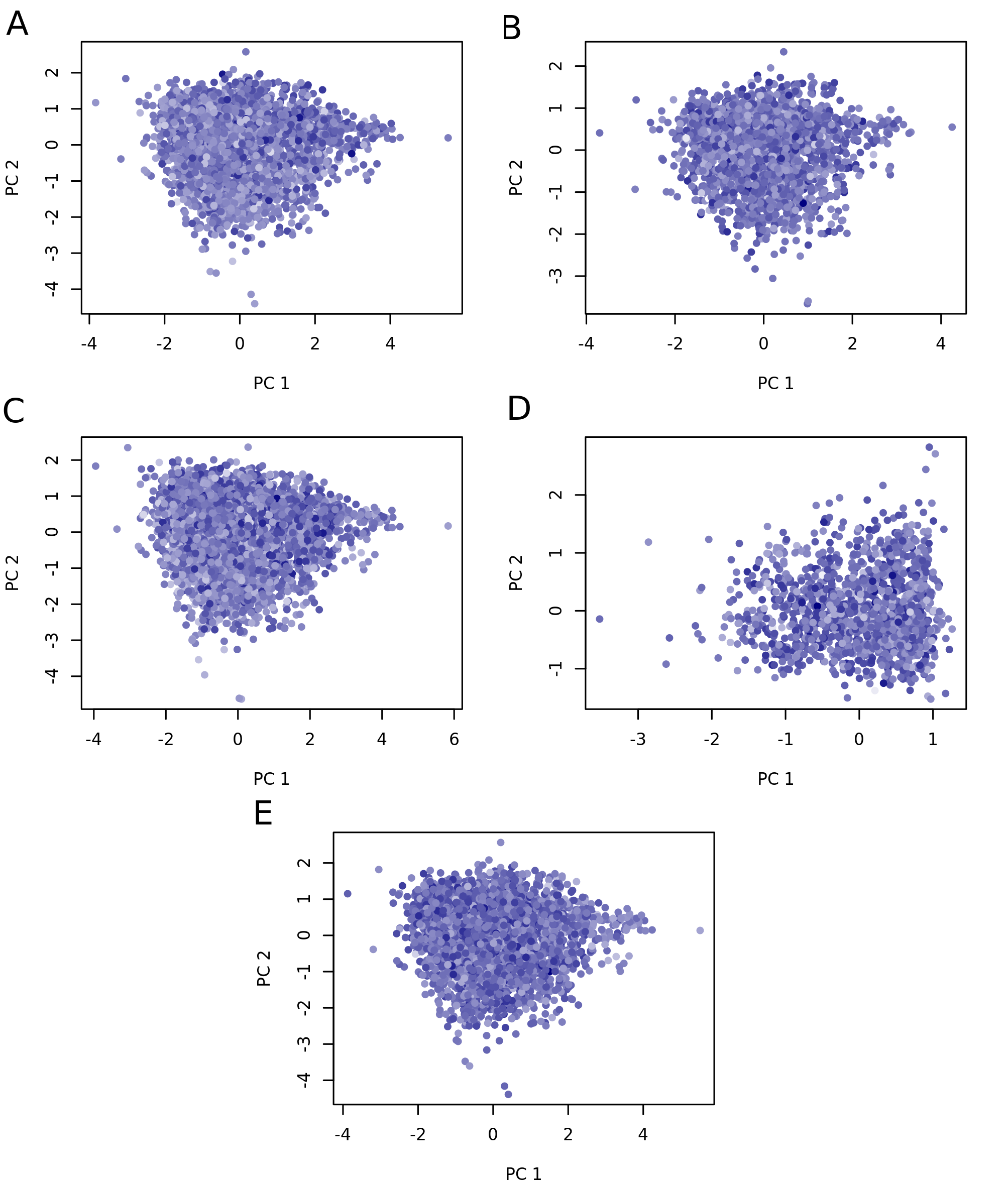

Supplement: Supplementary file 4 — The first two principal coponents for the genomic relationship matrix comprising individuals used for genome-wide association of fertility traits (A) Non-return rate in cows (B) Non-return rate in heifers (C) Interval from first to last insemination in cows (D) Interval from first to last insemination in heifers (E) Days to first service. (PNG 677 kb) [file 12864_2017_4308_MOESM4_ESM.png]

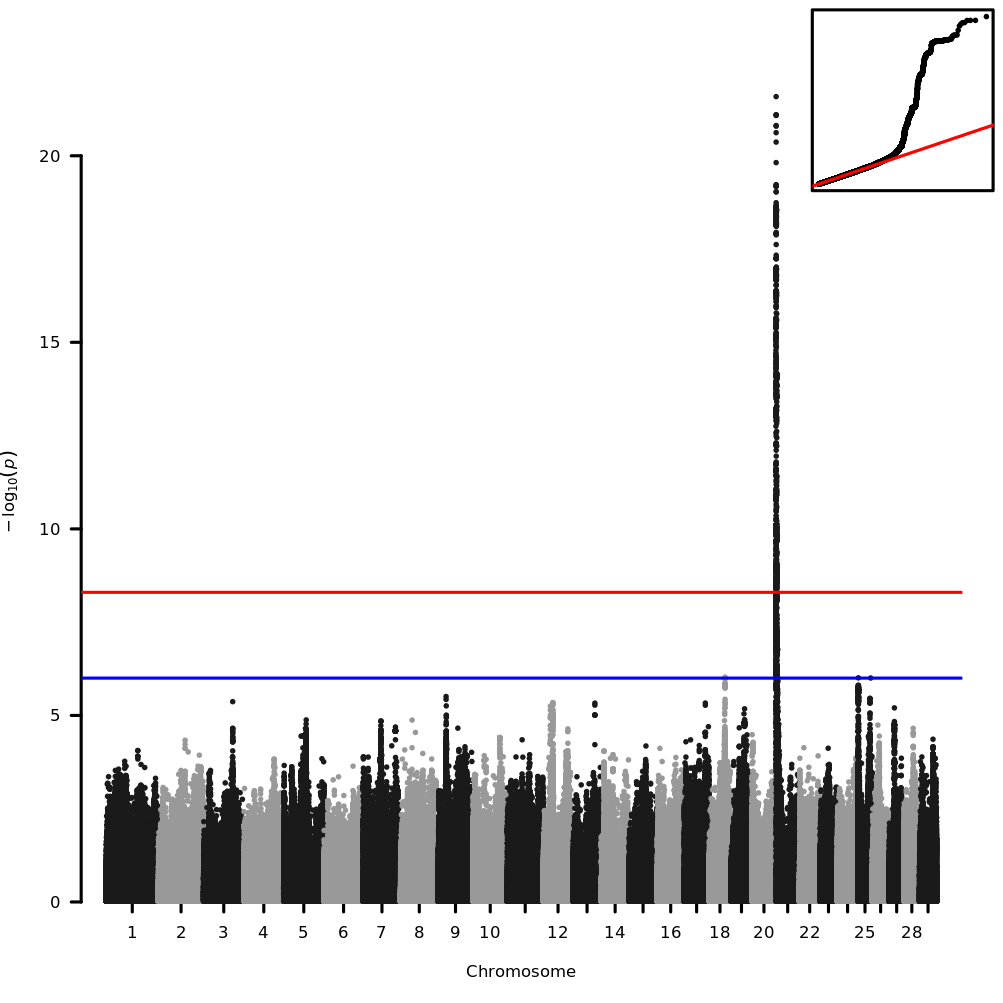

Supplement: Supplementary file 7 — GWAS for calving ease using stature as a covariate. The red line marks the Bonferroni corrected significance threshold. The blue line shows the threshold for suggestive variants. The figure in the upper right corner shows the corresponding qqplot. (PNG 74 kb) [file 12864_2017_4308_MOESM7_ESM.png]

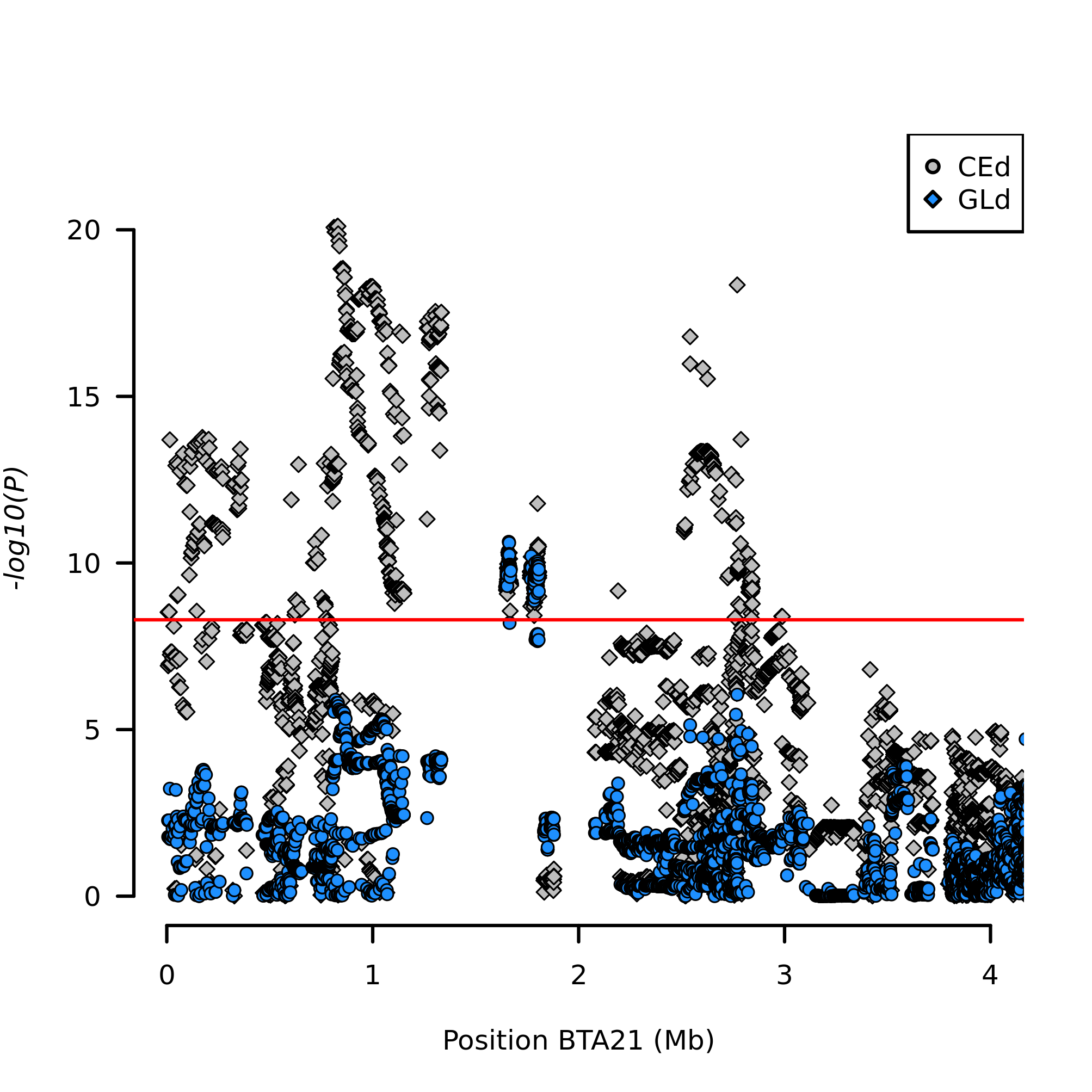

Supplement: Supplementary file 8 — Association for calving ease direct (CEd) and gestation length direct (GLd) on BTA21 from 1 to 4 Mb. The red line indicates the Bonferroni corrected significance threshold. (PNG 582 kb) [file 12864_2017_4308_MOESM8_ESM.png]

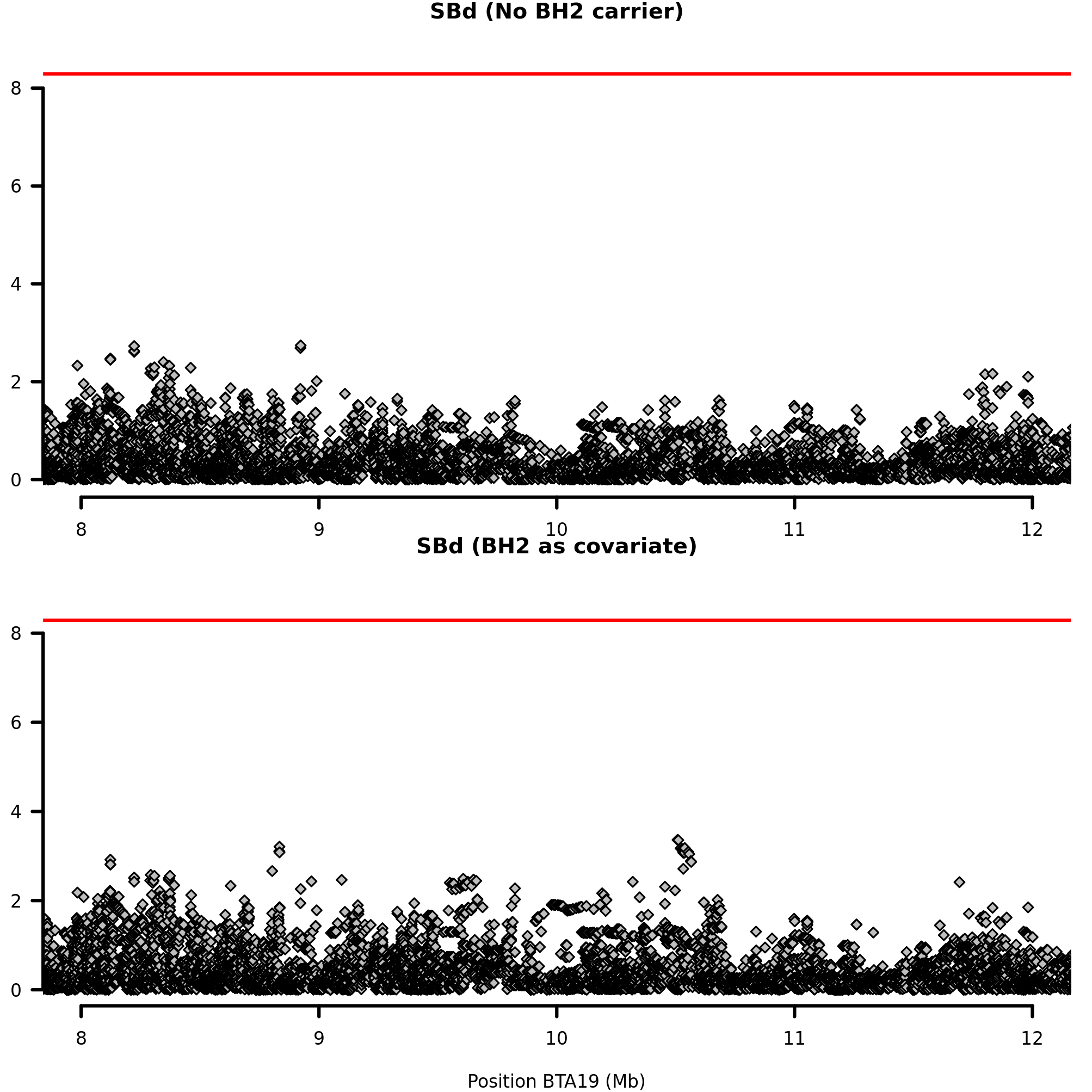

Supplement: Supplementary file 9 — Association on BTA19 for stillbirth using only individuals not carrying BH2 (top) or using BH2 carrier status as a covariate (bottom). The red line indicates the Bonferroni corrected significance threshold. (PNG 563 kb) [file 12864_2017_4308_MOESM9_ESM.png]

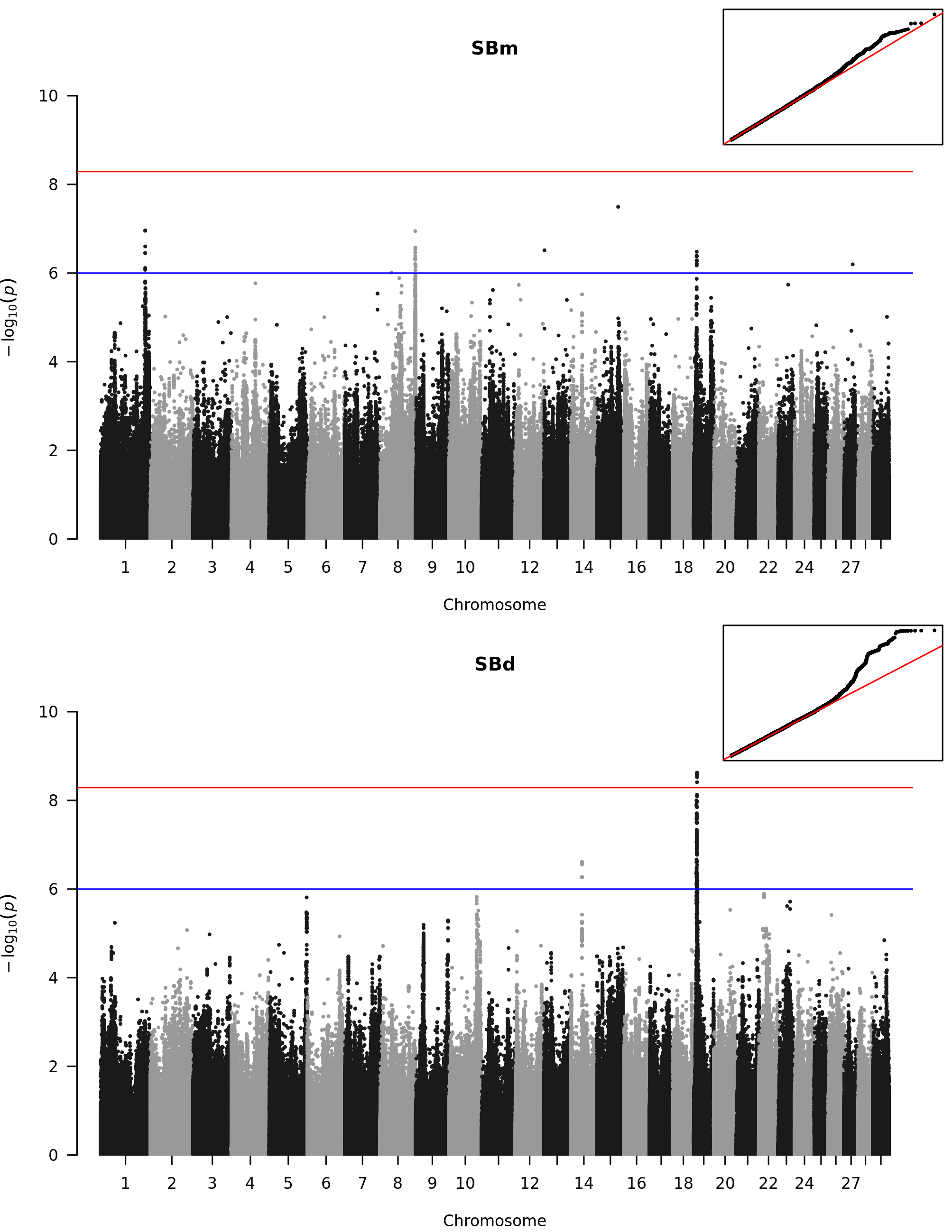

Supplement: Supplementary file 10 — Manhattan plots for the GWAS for stillbirth direct and maternal excluding individuals from the smaller cluster in the PCA plot. The red lines mark the Bonferroni corrected significance threshold. The blue lines show the threshold for suggestive variants. Small figures in the upper right corner shows the corresponding qqplot. (PNG 506 kb) [file 12864_2017_4308_MOESM10_ESM.png]
